# Commentary: Zika Virus in the Americas—Yet Another *Arbovirus* Threat

**DOI:** 10.3389/fmicb.2018.00435

**Published:** 2018-03-12

**Authors:** Giovanni Di Guardo

**Affiliations:** Faculty of Veterinary Medicine, University of Teramo, Teramo, Italy

**Keywords:** Zika virus, arthropod-borne viruses, mosquitoes, *Aedes aegypti*, *Aedes albopictus*, *Wolbachia* spp., *Dirofilaria immitis*

As highlighted in the nice and interesting article by Drs. Anthony S. Fauci and David M. Morens, the “anthropocentric” world we live in is characterized by a progressive demographic expansion and urban population's growth, coupled with a steadily increasing trend of people's and animals' travels and movements, as well as with human-induced ecological changes (Fauci and Morens, 2016). The latter are of special concern with reference to the “ecological niches” colonized by Zika virus (ZIKV) and other arthropod-borne pathogens, either flaviviral or non-flaviviral, like Yellow Fever virus (YFV), Dengue virus (DV), West Nile virus (WNV), and Chikungunya virus (CV) (Fauci and Morens, 2016). Indeed, apart from WNV, which is mainly carried by *Culex* spp., *Aedes* (*Ae*.) spp. mosquitoes, particularly *Aedes aegypti*, are primarily involved in YFV, DV, CV, and ZIKV interhuman transmission. Within such context, the role played by Asian tiger mosquitoes (*Aedes albopictus*) in ZIKV ecology, epidemiology and interhuman spread deserves special attention, being *Ae. albopictus* far more common than *Ae. aegypti* in the Northern and Western Hemispheres (Fauci and Morens, 2016; Gregory et al., 2017).

Among the many challenging and open issues regarding the biology of ZIKV infection and the vertebrate/invertebrate host-pathogen interaction(s) with such virus, the only flaviviral agent with a documented teratogenic potential (Fauci and Morens, 2016), the characterization of ZIKV neurotropism and neuropathogenicity is of paramount relevance and could greatly benefit from the use of suitable animal models (Di Guardo et al., 2016; Fauci and Morens, 2016).

Furthermore, employing *Wolbachia* spp.—a Gram-negative bacterial microorganism infecting only invertebrates—as a “natural weapon” against ZIKV (and YFV, DV, WNV, and CV) might represent a valuable option (Gulland, 2016; Waltz, 2017). In this respect, while it has been estimated that *Wolbachia* spp. could infect 60% of insects worldwide, a similar situation does not appear to be true—at least under natural conditions—for *Ae. aegypti*. Notwithstanding the above, *Wolbachia* spp. has been also indicated as an efficient tool against *Ae. albopictus* (Waltz, 2017), which is regarded as an additional ZIKV vector, albeit with a competence and transmission capacity lower than *Ae. aegypti* (Fauci and Morens, 2016; Gregory et al., 2017). Indeed, utilizing a “natural weapon” like *Wolbachia* spp. in the fight against *Ae. albopictus* could lead, among others, to a reduction in the use of chemical insecticides, that are known to undergo dangerous “bioaccumulation” and “biomagnification” processes along animal food chains (Aznar-Alemany et al., 2017).

Notably, *Ae. albopictus* is an efficient vector for *Dirofilaria immitis*, a canine and feline cardio-pulmonary nematode which may also be infected by *Wolbachia* spp. (Morchón et al., 2012). Since a *Wolbachia* spp.-induced inflammatory response may occur in *D. immitis*-affected dogs and cats (Frank and Heald, 2010), I believe it would be interesting to investigate “whether and how” interfering by means of *Wolbachia* spp. in the ecology, epidemiology and evolution of given arthropod-borne infections could affect not only the “mosquito vector” but also the “organism” carried inside it, be it a virus (as in the case of ZIKV) or a parasite (as in the case of *D. immitis*). In fact, antimicrobial therapy against *Wolbachia* spp. has been shown to result in decreased microfilarial loads, inhibition of the development of larval worms, female worm infertility and reduced numbers of *Wolbachia* spp. organisms (Frank and Heald, 2010). Consequently, at least in *D. immitis*-infected dogs and cats, the presence and the number of *Wolbachia* spp. organisms appear to get along with the infection's progression, quite differently from what may be expected to occur in ZIKV-infected, *Wolbachia* spp.-challenged mosquitoes (Gulland, 2016; Waltz, 2017).

Noteworthy, host's inflammatory responses can modulate pathogens' shedding and transmission dynamics, as clearly documented in *Streptococcus pneumoniae*-challenged mice (Zafar et al., 2017) and in *Scrapie*-infected sheep with lentiviral mastitis (Ligios et al., 2011).

In summary, we are dealing with a very complex and intricate “balance,” resulting from the mutual interaction(s) between the “pathogen,” either viral (ZIKV) or parasitic (*D. immitis*), the “mosquito vector,” the “host,” and the “environment,” with further investigation on these challenging and intriguing issues being absolutely needed.

## Author contributions

The author confirms being the sole contributor of this work and approved it for publication.

### Conflict of interest statement

The author declares that the research was conducted in the absence of any commercial or financial relationships that could be construed as a potential conflict of interest.
